# Lymphomas in cartilage-hair hypoplasia – A case series of 16 patients reveals advanced stage DLBCL as the most common form

**DOI:** 10.3389/fimmu.2022.1004694

**Published:** 2022-09-23

**Authors:** Hanna-Leena Kukkola, Pauliina Utriainen, Pasi Huttunen, Mervi Taskinen, Outi Mäkitie, Svetlana Vakkilainen

**Affiliations:** ^1^ Children and Adolescents, Pediatric Research Center, Children’s Hospital, University of Helsinki and Helsinki University Hospital, Helsinki, Finland; ^2^ Department of Pediatric Hematology, Oncology and Stem Cell Transplantation (SCT), Children‘s Hospital, University of Helsinki and Helsinki University Hospital, Helsinki, Finland; ^3^ Folkhälsan Research Center, Institute of Genetics, Helsinki, Finland; ^4^ Research Program for Clinical and Molecular Metabolism, Faculty of Medicine, University of Helsinki, Helsinki, Finland; ^5^ Department of Molecular Medicine and Surgery, Karolinska Institutet, Stockholm, Sweden; ^6^ Clinical Genetics, Karolinska University Hospital, Stockholm, Sweden

**Keywords:** Immunodeficiency, chemotherapy, chondrodysplasia, malignancy, cancer

## Abstract

**Background:**

Patients with cartilage-hair hypoplasia (CHH) have an increased risk of malignancy, particularly non-Hodgkin lymphoma and basal cell carcinoma. The characteristics, clinical course, response to therapy and outcome of lymphomas in CHH remains unexplored.

**Methods:**

We assessed clinical features of lymphoma cases among Finnish patients with CHH. Data were collected from the Finnish Cancer Registry, hospital records, the National Medical Databases and Cause-of-Death Registry of Statistics Finland.

**Results:**

Among the 160 CHH patients, 16 (6 men, 10 women) were diagnosed with lymphoma during 1953-2016. Lymphoma was diagnosed in young adulthood (median age 26.4 years, range from 6.4 to 69.5 years), mostly in advanced stage. The most common lymphoma type was diffuse large cell B-cell lymphoma (DLBCL) (6/16, 38%). Eight patients received chemotherapy (8/16, 50%), and two of them survived. Standard lymphoma chemotherapy regimens were administered in the majority of cases. Altogether, eleven CHH patients died due to lymphomas (11/16, 69%). In almost all surviving lymphoma patients, the diagnosis was made either during routine follow-up or after evaluation for non-specific mild symptoms. Search for CHH-related clinical predictors demonstrated higher prevalence of recurrent respiratory infections, in particular otitis media, and Hirschsprung disease in patients with lymphoma. However, three patients had no clinical signs of immunodeficiency prior to lymphoma diagnosis.

**Conclusion:**

DLBCL is the most common type of lymphoma in CHH. The outcome is poor probably due to advanced stage of lymphoma at the time of diagnosis. Other CHH-related manifestations poorly predicted lymphoma development, implying that all CHH patients should be regularly screened for malignancy.

## Introduction

Cartilage-hair hypoplasia (CHH) is caused by variants in the *RMRP* gene (1, 2) that result in a wide spectrum of manifestations including short stature due to metaphyseal chondrodysplasia, hair hypoplasia, abnormal erythropoiesis, and immune deficiency, (3–6). Hirschsprung disease (HSCR), and malignancies occur at increased frequency (7–10).

Previous studies have demonstrated significantly increased mortality in patients with CHH compared to their parents and non-affected siblings, with malignancies as the leading cause of death (11). In Finnish CHH patients, evaluated between 1967 and 1995, a sevenfold cancer risk was observed compared with the age-adjusted expected incidence (8). Non-Hodgkin lymphomas and basal cell carcinomas are the most prevalent types of cancer (12, 13). The standardized incidence ratio (SIR), which describes the observed number of malignancy cases in CHH divided by the expected number of cases derived from general population, was 90 for lymphomas and 33 for basal cell carcinomas. Most cancers were diagnosed in young adults aged from 15 to 44 years (12). Others have reported similar findings in non-Finnish patients with CHH (14–17).

The mechanism by which the *RMRP* variants in CHH predispose to malignancies is unknown. The pathogenic variants in the untranslated *RMRP* gene, a long non-coding RNA, disturb ribosomal processing (18, 19), leading to altered cytokine signaling and dysregulation of genes involved in cell cycle and cell growth control in terminally differentiated cells in lymphocytic and chondrocytic cell lines (18, 20). It has been speculated that some of these mechanisms may be involved in the pathogenesis of malignancies in CHH (18). In addition, telomere function is impaired in CHH, and it may contribute to the increased risk of malignancies (21, 22).

Lymphomas in CHH have been associated with poor prognosis. Out of 14 Finnish patients with diagnosis of cancer described previously, among them 10 with lymphoma, nine patients deceased and the median survival time after diagnosis was three months (12). To date no consensus exists on optimal treatment of patients with diagnosed immunodeficiency and lymphoma (23). The high prevalence, poor outcome and lack of evidence for the management of lymphoma prompted us to explore in detail the diagnosis, management, and course of lymphomas in patients with CHH in order to identify means to improve early detection and treatment outcome. We describe 16 lymphoma cases in the Finnish CHH cohort, search for correlates between clinical features and the development of lymphoma and address therapeutic and management options.

## Methods

The study population and patient data were collected from several Finnish National Medical Databases. Data on malignancies were collected from the Finnish Cancer Registry covering period from 1953 to 2016, and mortality data from the Cause of-death Registry of Statistics Finland from 1971 to 2016. Data from the Finnish National Care Registry for Health Care (HILMO) covered period from 1969 to 2016, and included data on inpatient health service providers, while the Finnish National Registry of Primary Health Care Visits (AVOHILMO) covered outpatient health service provider data from 2011 to 2016.

The Finnish Cancer Registry is population-based and covers the whole country since its foundation in 1952. Data is gathered from all hospitals, health care centers, pathologic and hematologic laboratories, forensic autopsies, and death certificates. The coverage is almost complete (99%) (24) since reporting is obligatory.

From the Finnish Cancer Registry, we identified lymphatic malignancy in 16 out of the 160 CHH patients that are included in the Skeletal Dysplasia Register (13). We then obtained these patients’ health records from all identified health service providers for further analysis. We collected information of the type, location and staging of lymphomas, treatment, lymphoma-free survival, and outcome. Also, we collected information of other diagnosed malignancies, previous infections, other signs of immunodeficiency, growth parameters, and immunologic laboratory indices, when available. The birth measurements and standard deviation (SD) values were corrected according to gestational age (25). When comparing the growth parameters at the time of lymphoma diagnosis to non-lymphoma patients we used the previously established CHH-specific growth charts (26).

When evaluating other clinical characteristics of CHH patients in correlation to lymphomas, we used the data of previously published well-characterized CHH patient cohort that included 71 non-lymphoma CHH patients (13).

The study was approved by the Ethics Review Board, Helsinki University Hospital (HUS/836/2018). Only registry data were used and therefore no patient consents were needed. However, three of the four surviving patients have been recruited into the study and consented.

The SIR for lymphatic malignancies were derived by dividing the number of observed lymphoma cases by the number of expected lymphomas in the general population.

For the assessment of correlations between lymphoma development and other clinical features, non-parametric statistical analysis of Chi-Square test of Independence, Mann-Whitney U -test and Kruskal-Wallis test, as well as multivariate regression analysis were used as appropriate.

P-values <0.05 were considered statistically significant. Statistical analyses were accomplished with the IBM SPSS Statistics (version 22-23, 25 and 27).

## Results

### Lymphomas in patients with CHH

Among the 160 Finnish patients with CHH, 16 had been diagnosed with lymphoma during the study period. **Table 1** and **2** demonstrate the characteristics of CHH patients with lymphoma. The lymphoma diagnoses were made between years of 1983 to 2012. Lymphoma was typically diagnosed in young adulthood (median age at diagnosis 26.4 years, range from 6.4 to 69.5 years), and was fatal in 11/16 patients (69%).

**Table 1:** Demographics of the study participants.

Clinical characteristics	Value
Sex, female/male, number of patients (n=16)	10/6
Age in years at the time of lymphoma diagnosis, median (IQR) (n=16)	26.4 (20.4 – 40.1)
Height in cm at the time of lymphoma diagnosis, median (IQR) (n=11)	126.0 (100.5 – 137.0)
Height in cm in adults, median (IQR) (n=9)	133.8 (123.5 – 137.8)
Height in cm in children, median (IQR) (n=2)	107.1 (88.6 – NA)
Height SD in children, median (IQR) (n=2)	-5.5 (-5.6 – NA)
CHH-specific height percentile*, median (IQR) (n=11)	50.0 (25.0 – 75.0)
Previous malignancies, number of patients (n=12)	1

*Derived from CHH-specific growth charts (5). CHH, cartilage-hair hypoplasia; IQR interquartile range; n, number of patients; NA, not applicable; SD, standard deviation.

**Table 2:** Description of lymphoid malignancies in study patients.

Case	Age group, years	Lymphoma type	Lymphoma location	TNM stage at diagnosis	Cancer Register staging*	Chemotherapy	Other treatment	Remission	Relapse	Outcome
1	40 - 50	B-cell Burkitt lymphoma	Intra-abdominal LN	Unknown	3	No, palliative care due to advanced lymphoma and sudden deterioiration	No	No	No	Fatal
2	20 - 30	MALT-lymphoma	Stomach	Unknown	Unknown	No	Antimicrobials for *Helicobacter*	Yes	No	Alive
3	40 - 50	DLBCL (centroblastic variant)	Bone marrow	IV	3	R-CHOP	No	Unknown	Unknown	Fatal
4	20 - 30	DLBCL	Spleen	III	Unknown	R-CHOP x 6 +, Rituximab monotherapy for 12 months	No	Yes	No	Alive
5	<20	DLBCL	LN	Unknown	3	No	Unknown	No	No	Fatal
6	30 - 40	ALCL	Neck lymphoid tissue	Stage IA, IPI-score 1	1	CHOP, ESHAP	Radiation therapy, PUVA-treatment	No	Yes	Fatal
7	30 - 40	CLL, Richter’s transformation	Bone marrow and ovarium	Unknown	3	CHOP x 6, Fludara x 2	No	No	No	Fatal
8	30 - 40	DLBCL	Lungs and abdominal cavity	IVA, (relapse IVB)	Unknown	CHOP x 8, ESHAP x 2 reducted; in relapse R-CHOP + MTX, R-HD-MTX-cytarabin x 3, R-CHOP-MTX x 2	No	Yes	Yes	Alive
9	20 - 30	nHL, NOS	Small intestine	Unknown	3	No, due to patient’s refusal	No	No	No	Fatal
10	40 - 50	nHL, NOS	Intra-abdominal LN	IVB	4	M-BACOD, DHAP	No	No	No	Fatal
11	<20	nHL, NOS	LN	IV	3	Unknown	Unknown	Unknown	Unknown	Fatal
12	20 - 30	NSHL	Lungs	IIIB	Unknown	BEACOPP	No	No	No	Death of another cause
13	20 - 30	DLBCL	Unknown primary cite	Unknown	Unknown	Unknown	Unknown	Unknown	Unknown	Fatal
14	20 - 30	Spindle cell cancer	Mediastinum	Unknown	1	ALL high risk	Radiation therapy	No	No	Fatal
15	20 - 30	DLBCL	Liver	Unknown	3	Unknown	Unknown	Unknown	Unknown	Alive
16	>50	Plasma cell myeloma	Bone marrow	Unknown	3	Unknown	Unknown	Unknown	Unknown	Fatal

ALCL, Anaplastic large cell lymphoma; ALL, Acute lymphocytic leukeamia; BEACOPP, bleomycin, etoposide, doxorubicin hydrochloride, cyclophosphamide, vincristine, procarbazine, prenisone; CHOP, cyclophosphamide, doxorubicin hydrochloride, vincristine, sulfate, prednisone; CLL, Chronic lymphocytic leukemia; DHAP, dexamethasone, high-dose cytarabine, cisplatin; DLBCL, Diffuse large B-cell lyhmphoma; ESHAP, etoposide, methylprednisolone, cytarabine, cisplatin; Fludara, fludarabine cytostatic treatment; LN, lymph node; MALT-lymphoma, mucosa-associated lymphoid tissue lymphoma; M-BACOD, methotrexate, bleomycin, doxorubicin, cyclophosphamide, vincristine, dexamethasone; MTX, methotrexate; nHL, non-Hodgkin lyphoma; NOS, not otherwise specified; NSHL, Nodular sclerosing Hodgkin lymphoma; PUVA-treatment, Psoralen and UVA ultraviolet light therapy treatment; R-CHOP, rituximab, cyclophosphamide, doxorubicin hydrochloride, vincristine, sulfate, prednisone; R-HD-MTX-cytarabin, rituximabe, high-dose methotrexate, cytarabin treatment; TNM stage, is a classification of malignant tumors where T describes the size of the original (primary) tumor and whether it has invaded nearby tissue, N describes nearby (regional) lymph nodes that are involved and M describes distant metastasis.

*Before lymphoma diagnosis the patient had cervical dysplasia.

*Classified as follows: 1 Localized, 3 Metastasized beyond regional lymph nodes or into adjacent tissues, 4 Metastasized, no data on extent.

The most common lymphoma types were diffuse large cell B-cell lymphoma (DLBCL) (6/16, 38%), and other unspecified non-Hodgkin lymphomas (NHL, NOS) (3/16, 19%). Other types of lymphoma occurred as single cases (**Table 2**). Lymphoma was advanced at the time of diagnosis in most cases (TNM stage IV in four patients andIII in two patients). Three patients with stage IV disease had extranodal involvement in bone marrow, liver, or lungs; for one patient with stage IV lymphoma, the information on exact disease extent was not available. The median age at the time of death was 40.4 years (IQR 20.2 – 46.5 years). In total four patients were alive and disease free (4/16, 25%) at the end of study period, eleven patients deceased due to lymphomas (11/16, 69%), and one patient deceased of a lymphoma-unrelated cause (1/16, 6%). The median time from diagnosis to death was 104 days (IQR 25 – 887 days). In almost all surviving lymphoma patients, the diagnosis was made either during a scheduled follow-up visit or after evaluation for non-specific mild symptoms.

Epstein barr virus (EBV) was assessed in three patients and detected in one patient’s tumor of DLBCL with 6500 copies per milliliter. One patient was EBV positive in skin sample from the primary tumor cite of ALCL, but further analysis from samples taken later were negative. Another patient with DLBCL had chronic EBV viremia. Altogether, an association with EBV was thus observed in all three patients for whom the data on EBV testing were available.

Half of the patients received chemotherapy (8/16, 50%). Two of these patients were also treated with local radiotherapy. One patient with B-cell Burkitt lymphoma received palliative treatment because of advanced stage of lymphoma and sudden deterioration and one patient with MALT-lymphoma received antimicrobial therapy due to *Helicobacter pylori* infection. Another patient refused chemotherapy in favor of holistic treatment options and deceased two months after diagnosis. For three patients the information regarding the treatment was unavailable. None of the patients received stem cell transplantation either before or after the lymphoma diagnosis. The chemotherapy regimens are detailed in **Table 2**. Most of the patients received standard chemotherapy used in concurrent lymphoma treatment protocols, and only for one patient reduction of second line chemotherapy was applied as a precaution due to concern of worsening immunodeficiency. In patients who received chemotherapy, prophylactic antibiotic treatment with sulfa-trimethoprim was used. In seven patients for whom chemotherapy was given and details on regimens were available, three had received rituximab and two of them survived, compared to fatal outcome in all four patients whose regimens did not contain rituximab.

The Finnish Cancer Registry data demonstrated SIR of 34 (95% CI 17 – 60) for lymphoid and hematopoietic tissue malignancies in patients with CHH. During the study period The Finnish Cancer Registry changed their malignancy categorization, thus the SIR reported in the prior Finnish study of Taskinen et al. (12) cannot be compared to the SIR of current study.

### Comparison of clinical characteristics in CHH patients with and without lymphoma

Clinical characteristics of the CHH patients diagnosed with lymphoma were available for 11 individuals and are described in **Table 3**. All twelve patients with lymphoma with genotype data available had n.71A>G *RMRP* variants either in homozygous (n=7) or compound heterozygous (n=4 for n.263G>T and n=1 for dup TACTCTGTGA at -13 variants) forms. Patients demonstrated remarkable variability in clinical and laboratory manifestations of immunodeficiency. Severe or opportunistic infections were common (5/11, 45%), however, three patients had no clinical signs of immunodeficiency documented prior to lymphoma diagnosis. Data on laboratory immunologic parameters were poorly available, but of note, four patients had normal total lymphocyte counts. However, only one of these four non-lymphopenic patients had all other laboratory immunophenotyping features in the normal range, while others had decreased lymphocyte proliferative responses. Consistent with previous reports in Finnish CHH population (7), 88% (7/8) of patients who have been tested for lymphocyte proliferation responses, demonstrated decreased responses to phytohemagglutinin.

**Table 3:** Characteristics of immunodeficiency in the 16 study patients.

Case	*RMRP* variants	Clinical signs of immunodeficiency	TLC	CD3+	CD4+	CD8+	CD19+	CD16/56+	Neutrophil count	Lymphocyte proliferation response to PHA	IgA	IgM	IgG	IGRT
1	n.71A>G/n.263G>T	None	2.46	NA	NA	NA	NA	NA	5.49	Decreased	**0.76**	0.50	13.0	No
2	n.71A>G/n.71A>G	Rec OM*	**1.15**	**0.66**	0.45	**0.17**	0.14	0.28	3.30	Decreased	1.43	1.23	13.0	No
3	NA	AI, Rec OM, Sin, Pn*	2.10	NA	NA	NA	NA	NA	3.72	Decreased	1.06	**0.08**	12.6	Yes
4	n.71A>G/n.71A>G	AI, Rec OM, Sin, Pn, and sepsis	**0.26**	**0.16**	**0.12**	**0.04**	**0.00**	**0.05**	**0.67**	Decreased	**0.46**	**0.20**	9.1	Yes
5	NA	NA	NA	NA	NA	NA	NA	NA	NA	NA	NA	NA	NA	NA
6	n.71A>G/n.71A>G	AI, Severe varicella**	**0.86**	NA	NA	NA	NA	NA	3.10	Decreased	3.82	**0.33**	**6.0**	No
7	n.71A>G/n.263G>T	None	1.95	NA	NA	NA	NA	NA	4.09	Decreased	1.50	0.77	14.6	No
8	n.71A>G/n.71A>G	Rec OM, refractory warts*	1.73	1.29	1.05	0.23	0.16	0.11	4.36	Normal	3.67	1.41	15.7***	No
9	NA	NA	NA	NA	NA	NA	NA	NA	NA	NA	NA	NA	NA	NA
10	n.71A>G/n.71A>G	NA	NA	NA	NA	NA	NA	NA	NA	NA	NA	NA	NA	NA
11	n.71A>G/dup TACTCTGTGA at -13	Rec OM*	NA	NA	NA	NA	NA	NA	NA	NA	NA	NA	NA	No
12	n.71A>G/n.71A>G	None	**1.19**	NA	NA	NA	NA	NA	4.14	NA	NA	NA	NA	No
13	n.71A>G/n.263G>T	Rec OM, Pn, disseminated EBV*	NA	NA	NA	NA	NA	NA	NA	NA	NA	NA	NA	Yes
14	n.71A>G/n.263G>T	Rec OM, Sin, Pn*, severe varicella**	**1.22**	1.21	**0.41**	0.53	0.12	0.20	2.15	Decreased	1.00	1.60	17.1	Yes
15	NA	NA	NA	NA	NA	NA	NA	NA	NA	NA	NA	NA	NA	NA
16	n.71A>G/n.71A>G	NA	NA	NA	NA	NA	NA	NA	NA	NA	NA	NA	NA	NA

AI, autoimmunity; EBV, Epstein-Barr virus; Ig, immunoglobulin; IGRT, immunoglobulin replacement therapy; NA, not available; OM, otitis media; PHA, phytohemagglutinin; Pn, pneumonia; Rec, recurrent; Sin, rhinosinuitis; TLC, total lymphocyte counts.

*Recurrent pneumonia: ≥2 episodes within a year or as ≥3 episodes ever; Recurrent OM or Sin: ≥3 episodes within 6 months, ≥ 4 within a year or ≥ 10 ever; Refractory warts: warts persisting for years and requiring multiple treatment courses.

**Severe varicella was defined as varicella requiring hospitalization.

***In addition, this patient demonstrated normal antibody responses to polysaccharide pneumococcal vaccine, for other patients vaccine response data were unavailable. Cell counts are reported as cells x 10^9^/L, immunoglobulin levels as g/L, and numbers in bold indicate decreased counts or levels according to the local laboratory reference values.

The comparison of clinical manifestations in CHH patients with and without lymphoma is presented in **Table 4**. We included only patients with timeline data available in the analysis of risk factors for lymphoma development. In a univariate analysis, recurrent sinopulmonary infections, and in particular, recurrent otitis media, were more frequent prior to the diagnosis of lymphoma. In the multivariate analysis, only recurrent otitis media was significantly associated with the development of lymphoma (OR 5.6, 95% CI 1.4 – 22.4, p=0.02).

**Table 4:** Comparison of clinical characteristics in CHH patients with and without lymphoma.

	CHH patients with lymphoma (n=11)	CHH patients without lymphoma (n=65)	p value of Mann-Whitney U test	OR (95% CI)
Median height, cm (IQR)	126.0 (100.5 – 137.0)	125.0 (118.0 – 135.0)	0.83	NA
Median height, percentile (IQR)	50.0 (25.0– 75.0)	50.0 (25.0 – 75.0)	0.20	NA
Birth SD, median (IQR)	-2.8 (-4.3 – (-2.3)	-3.0 (-3.5 – (-1.8)	0.67	NA
	CHH patients with lymphoma	CHH patients without lymphoma	Chi square χ^2^ (p value)	OR (95% CI)
Compound heterozygosity for pathogenic *RMRP* variants	5/12 (42%)	15/65 (23%)	1.82 (0.28)	2.38 (0.66-8.60)
Immunoglobulin replacement therapy	4/11 (36%)	4/71 (6%)	3.04 (0.11)	3.89 (0.77 – 19.35)
Hirschsprung disease	3/16 (19%)	3/71 (4%)	4.29 (0.07)	5.23 (0.95 – 28.83)
Autoimmunity	3/10 (30%)	7/71 (10%)	3.29 (0.10)	3.92 (0.82 – 18.67)
Recurrent sinopulmonary infections	7/10 (70%)	25/71 (35%)	4.44 (<0.05)	4.29 (1.02 – 18.08)
Recurrent pneumonia	3/10 (30%)	9/71 (13%)	2.095 (0.16)	2.95 (0.64 – 13.53)
Recurrent otitis media	6/10 (60%)	15/71 (21%)	6.90 (0.02)	5.60 (1.40 – 22.43)
Recurrent rhinosinusitis	3/10 (30%)	15/71 (21%)	0.40 (0.69)	1.60 (0.37 – 6.94)
Severe varicella	2/10 (20%)	2/71 (3%)	5.51 (0.07)	8.63 (1.06 – 69.89)
Refractory warts	0/10 (0%)	9/71 (13%)	1.433 (0.36)	0.87 (0.80 – 0.95)

CHH, cartilage-hair hypoplasia; CI, confidence interval; IQR, interquartile range; n, number; NA, not applicable; OR, odds ratio; SD, standard deviation.

None of the growth parameters (birth length or height at the time of lymphoma diagnosis, either in centimeters, as SD scores or CHH specific growth percentiles) differed significantly between CHH patients with and without lymphoma (**Tables 1** and **4**).

Out of 16 lymphoma patients three (19%) had confirmed Hirschsprung’s disease (HSCR) necessitating surgery during the first year of life, compared to 4.2% (3/71) prevalence in patients with CHH and no lymphoma and to the overall 10% incidence of HSCR in CHH (7, 27) (OR 5.23, 95% CI 0.95 – 28.83, p=0.07).

Among the CHH patients with lymphoma, four individuals received immunoglobulin replacement therapy before lymphoma diagnosis (4/16, 25%) compared to four out of 71 CHH patients without lymphoma (4/71, 5.6%) (Chi square χ2 = 3.04, p= 0.11). All but one of these four patients had advanced lymphoma which led to death soon after diagnosis (survival between 14 days and 3.5 months).

### Illustrative cases

#### Case 1. (patient 8)

This patient with CHH presented at 32 years of age with abdominal pain, headache, and visual impairment. An emergency cranial MRI scan showed signs of posterior reversible encephalopathy syndrome. A whole-body CT scan revealed parenchyma infiltration in both lungs, liver and pancreas, pleura effusion, peritoneal carcinosis, and tumor mass on the uterus and on both adnexes. The diagnosis was confirmed with liver biopsy as DLBCL, stage IVA. No bone marrow infiltration was detected.(**Table 2**)

Treatment was started with standard chemotherapy of cyclophosphamide, doxorubicin hydrochloride, vincristine, sulfate, prednisone (CHOP) (**Table 2**). After eight cycles of therapy the CT scan showed infiltration still being present in the liver, and the treatment was continued with two cycles of reduced etoposide, methylprednisolone, cytarabine and cisplatin. The patient then remained in remission until a relapse 15 years later, presented as enlarged lymph nodes on both armpits and a subcutaneous lump on the back. A whole-body and cranial CT-scan showed tumor mass on the lower back, and metastatic changes in lungs, kidneys, liver, pancreas, ventricle, dura mater and subcutaneously. Enlarged lymph nodes were on the left sub clavicular space and on the para-aortic space. Histological diagnosis was DLBCL, stage IVB. Fluorescence *in situ* hybridization of the tumor tissue was negative for *BCL6*, *C-MYC* and *BCL2* gene translocations, as well as for *p53* gene deletion.

The relapse was treated with two cycles of rituximab, CHOP and methotrexate, and three cycles of rituximab, high-dose methotrexate and cytarabin, and radiotherapy. A complete remission has continued for five years.

This patient was also surgically treated for squamous cell carcinoma, Morbus Bowen and spinocellular carcinoma before and after lymphoma relapse. Interestingly, common laboratory immunologic parameters (immunoglobulin A, M and G levels, total neutrophil, lymphocyte and lymphocyte subset counts, lymphocyte proliferative responses and antibody responses to pneumococcal polysaccharide vaccine) were in the normal range.

#### Case 2. (patient 4)

This patient has been followed regularly for CHH, HSCR and short bowel syndrome. The patient has also manifested an episode of autoimmune hemolytic anemia and multiple infections including catheter-associated bloodstream infections, recurrent pneumonias and middle ear infections. The patient receives immunoglobulin replacement therapy and trimethoprim prophylaxis for lymphopenia.(**Table 2**)

During a scheduled screening abdominal ultrasound at 20 years of age, a solid tumor was detected in the spleen. A biopsy of the tumor revealed DLBCL. Disease was staged as III. The patient received chemotherapy by standard protocol of six cycles of rituximab and CHOP and rituximab treatment for a year after that. The patient has been in remission for 11 years.

## Discussion

Our study is the first large case series of CHH patients diagnosed with lymphoma. We demonstrated that lymphoma was typically diagnosed in young adults (median age 26.4 years) at advanced stage. The most common lymphoma type was DLBCL. Patients in our lymphoma cohort had high prevalence of severe and opportunistic infections, and often received immunoglobulin replacement therapy, compared to CHH patients without lymphoma. However, several patients had no increased incidence of infections and had normal total lymphocyte counts, although advanced immunophenotyping was not available. Despite a dismal prognosis of lymphoma in CHH, some patients respond to therapy promptly and enter long remission even with stage IV lymphoma. Our study emphasizes the importance of regular follow-up of CHH patients, including scheduled abdominal ultrasound.

In our study the prognosis of CHH-related lymphoma was poor: 11 patients out of 16 (69%) died of the disease. Among the deceased, three had DLBCL. DLBCL is a clinically aggressive NHL, and it is the single most common lymphoma type in Western countries, accounting for over 30 percent of new diagnoses (28). It typically has good response to chemotherapy (29). In the general population, the prognosis of DLBCL is dependent of patient age, with excellent 3-year event-free survival of up to 79 percent in young adults (<60 years) with the current standard immune-chemotherapy treatment with rituximab-CHOP (29). Even before rituximab era, the survival exceeded 50 percent in this age group. In the older patients, aged over 60 years, a 5-year event -free survival of 59 percent was achieved with rituximab-CHOP treatment (29). Since its initially reported single agent-activity in indolent lymphomas in 1997, the role of rituximab has expanded to cover both indolent and aggressive lymphomas (30). Our current cohort includes patients diagnosed and treated from 1983 to 2012, that is, before and after rituximab era. Although the number of cases is too small to draw solid conclusions, 67% (2/3) of patients who had received rituximab, survived, compared to 0% (0/4) patients who had not received rituximab. Despite of this it seems that the outcome of DLBCL in CHH patients is worse than in the general population. Since our CHH patients were treated with concurrent standard chemotherapy regimens the differences in the outcome may be related to the biology of the disease or to the immunodeficiency of the CHH patients. Indeed, the epidemiology of lymphoma in CHH differs from general population: the median age at DLBCL diagnosis is in general approximately 65 years while CHH patients in our cohort presented with lymphoma mainly during early adulthood. DLBCL comprises of several molecular and biological subtypes with variable behavior and outcomes (31). Unfortunately, we do not have the information regarding molecular features of the DLBCL – or other lymphomas – in our current retrospective cohort, apart from a single negative test for *BCL6*, *C-MYC*, *BCL2* and *p53* gene defects. The unavailable molecular/cytogenetics data makes it difficult to dissect whether the poor survival is unique to CHH or is due to adverse molecular characteristics of the lymphoma. The exact role of RMRP in the development of malignancy is unknown and further genotype-phenotype, as well as lymphoma endotype investigations are warranted.

No consensus exists on how to treat patients with impaired immune system with cancer diagnosis and especially with lymphoma. For example, previously HIV patients experienced significant toxicity and shorter remissions on chemotherapy related to their advanced HIV infection (32, 33). Thus, reduced-intensity chemotherapy became the standard of care, but this approach is now obsolete with current antiretroviral therapy (33). Interestingly, CHH patients in our cohort were treated with concurrent standard protocols, and previous history of immunodeficiency did not alter the chemotherapy given, except in a single patient. Still only few of the cohort patients had severe infections during the chemotherapy and none of them died of therapy-related infections. This may be partly because of short overall median survival in some patients with aggressive disease, or due to the clinically mild immunodeficiency in Finnish patients with CHH. However, this may also suggest that selected CHH patients with lymphoma can be treated with effective standard treatment protocols without routinely reducing dosages or intensity of chemotherapy.

Chimeric antigen receptor T-cell (CAR-T) therapy is a novel and promising cell therapy for relapsing and refractory B-cell malignancies (34). Promising results and long-term remissions have also been obtained using CAR-T cells in B-cell lymphomas, including Burkitt lymphoma and DLBCL (35). This therapy might be a good option or even a front-line therapy for CHH patients in the future to improve poor outcome.

CHH patients often suffer from recurrent upper and lower respiratory tract infections, including otitis media, sinusitis and pneumonia (36, 37). In this study cohort, seven patients had recurrent sinopulmonary infections before lymphoma diagnosis, and recurrent otitis media arose as a risk factor for lymphoma development. The small sample size of our cohort prompts for cautious interpretation of this association. Most importantly, several patients did not manifest any clinical signs of immunodeficiency prior to the diagnosis of lymphoma, which calls for regular malignancy screening of all, also asymptomatic, CHH patients. In addition, advanced immunophenotyping, particularly B cell subset counting, is warranted to further evaluate the association of lymphoma development and immunologic profile.

In the majority of cases, CHH in Finnish individuals develops due to n.71A>G *RMRP* variants in homozygous or compound heterozygous forms. This narrow genotype makes studies on genotype-phenotype correlations challenging. Although duplications in *RMRP* have been previously shown to associate with less severe short stature (38), the youngest patient in our cohort (also reported in the study of Klemetti, et al) with fatal lymphoma had a genotype of n.71A>G/dup TACTCTGTGA at -13. With the development of potential tools of predicting the pathogenicity of *RMRP* variants (39), future studies should explore risk factors for severe course in CHH, including both genetic and detailed immunophenotype data that were absent for many patients in our cohort. Although laboratory immunologic indices do not always correlate with the severity of immunodeficiency in CHH (40), advanced immunologic profiling, including T and B cell subset counting, should be collected in future studies (41, 42).

HSCR is a well-recognized co-morbidity in patients with CHH. It demonstrates a more severe clinical course and poor prognosis in CHH (11, 43). Prior data confirmed this association and linked HSCR with mortality in subjects with CHH (OR 7.2, 95% CI 1.04 – 55, p<0.01) (13). Cases of CHH with HSCR may represent severely abnormal *RMRP* function resulting in a more profound immunodeficiency. HSCR has not been associated with specific genotype in CHH, nor has the outcome of hematopoietic stem cell transplant in CHH patients with HSCR been reported. In our study cohort three patients had confirmed diagnosis of HSCR, and of these patients two had a fatal outcome after lymphoma diagnosis. The one patient who survived had an early diagnosis of lymphoma due to scheduled routine abdominal ultrasound. Thus, it should be acknowledged that CHH patients with HSCR have more severe disease course and might have higher risk for malignancies including lymphomas.

None of our patients received stem cell transplantation. It has been arbitrated that CHH patients with severe immunodeficiency should be considered for stem cell transplantation due to the high risk of NHL (12). Whether the development of lymphoma could be prevented with transplantation in CHH patients with or without signs of immunodeficiency, remains to be evaluated in the future. More knowledge on this can accumulate when transplanted pediatric CHH patients reach adulthood. We have reported here EBV positivity in all three patients for whom EBV testing has been performed. In addition, rituximab seemed to be an effective addition to therapy in our cohort. It is, therefore, tempting to speculate on the benefit of early rituximab therapy or even hematopoietic stem cell transplantation as preventive management strategy in CHH patient with chronic EBV viremia.

We recognize several limitations in this study. A significant part of data was collected from registries and health records. Thus, we cannot ensure the completeness of the obtained data, but the use of Finnish National Health Registries allowed excellent data accuracy and coverage (44). We indeed had missing information related to some of the variables, including detailed immunophenotyping, which might affect the validity of the statistical analyses. The unavailable data on chemotherapy may confound the survival data. We recognize that the sample size was small with only 16 lymphoma patients. However, the study cohort is the largest described in CHH patients with lymphoma and yields valuable information to clinicians treating CHH patients.

## Conclusions

CHH patients have markedly increased risk of lymphomas. The prognosis has remained poor due to partly unknown pathogenetic mechanisms and advanced stage of lymphoma at the time of diagnosis. CHH patients need to be screened regularly keeping in mind the high risk for malignancies and undergo a thorough diagnostic work-up even for mild symptoms. Further studies to elucidate risk factors for the development of malignancy in CHH should include detailed immunophenotype and international patients to expand the genotype data.

## Data availability statement

The original contributions presented in the study are included in the article/supplementary material. Further inquiries can be directed to the corresponding author.

## Ethics statement

The studies involving human participants were reviewed and approved by Ethics Review Board, Helsinki University Hospital. Written informed consent for participation was not provided by the participants’ legal guardians/next of kin because: Not required for this type of study (Registry Study).

## Author contributions

PU, PH, MT, OM and SV designed the study project. PU, SV and H-LK collected data from medical records. SV and H-LK performed statistical analysis. All authors contributed to the article and approved the submitted version.

## Funding

Outi Mäkitie’s research was supported by Sigrid Jusélius Foundation, Academy of Finland, Novo Nordisk Foundation, Folkhälsan Research Foundation, and the Swedish Childhood Cancer Foundation. Pauliina Utriainen’s research was supported by the Foundation of Pediatric Research, Väre Foundation for Pediatric Cancer Research and Helsinki University Research Funds.

## Acknowledgments

We thank our research team and especially our research nurse Nea Boman for her invaluable help with data collection and patient communication.

## Conflict of interest

The authors declare that the research was conducted in the absence of any commercial or financial relationships that could be construed as a potential conflict of interest.

## Publisher’s note

All claims expressed in this article are solely those of the authors and do not necessarily represent those of their affiliated organizations, or those of the publisher, the editors and the reviewers. Any product that may be evaluated in this article, or claim that may be made by its manufacturer, is not guaranteed or endorsed by the publisher.

## Abbreviations

CHH, Cartilage hair hypoplasia; CHOP, Chemotherapy of cyclophosphamide, doxorubicin hydrochloride, vincristine, sulfate, prednisone; DLBCL, Diffuse large cell B-cell lymphoma; EBV, Ebstein barr virus; HSCR, Hirschsprung’s disease; NHL, Non-Hodgkin lymphomas; SIR, Standardized incidence ratio.
